# 

**Published:** 1906-09-15

**Authors:** 


### CONTENTS.

Event and Comment - - - - - - - - - 433

Missing Teeth in Their Relation to Orthodontia and Bridge-work, By Milton T. Watson, D.D.S. 437

President's Address, - - - - - By J. J. Green, D.D.S. 446

The Use of the Pyrometer in Fusing Porcelain, By R. M. Pelton, D.D.S. 450

The Fineness of Gold Solders, By S. H. Guilford, A.M., D.D.S., Ph.D. 458

With Our Editors - - - - - - - - - - 463

Indents - - - - - - - - - - - - 473

Announcements - - - - - - - - - - 484

